# Acute pancreatitis and vasoplegic shock associated with leptospirosis – a case report and review of the literature

**DOI:** 10.1186/s12879-019-4040-1

**Published:** 2019-05-08

**Authors:** Alexander Maier, Rafael Kaeser, Robert Thimme, Tobias Boettler

**Affiliations:** 1 Department of Cardiology and Angiology I, Heart Center Freiburg University, Faculty of Medicine, University of Freiburg, Freiburg, Germany; 2 Department of Medicine II, Medical Center, Faculty of Medicine, University of Freiburg, Hugstetter Strasse 55, 79106 Freiburg, Germany

**Keywords:** Leptospirosis, Pancreatitis, Septic shock, Europe, Case report

## Abstract

**Background:**

Leptospirosis or Weil’s disease is caused by pathogenic spirochete bacteria called *Leptospira.* It is considered the most common zoonosis in the world and is usually transmitted by urine of rodents and dogs with an incubation time of 7–14 days. The clinical spectrum ranges from a subclinical infection to a fulminant septic course.

**Case presentation:**

Here, we report the case of a German patient with acute pancreatitis associated with *Leptospira interrogans* causing fulminant septic shock. The patient was successfully treated with intravenous antibiotics and left the hospital fully recovered after 18 days.

**Conclusions:**

To our knowledge, this is the first case of leptospirosis with acute pancreatitis as the leading clinical manifestation in Central Europe. Serologic and molecular genetic tests for leptospirosis should be considered, if no other causes for pancreatitis can be identified.

## Background

Leptospirosis or Weil’s disease is caused by pathogenic spirochete bacteria called *Leptospira.* It is considered to be the most common zoonosis worldwide and is usually transmitted from rodents, dogs and cats when an infected animal or an animal’s urine comes into contact with broken skin. The incubation time is 7–14 days. Absence of infected skin makes the entry unidentifiable in most cases. The clinical spectrum ranges from a subclinical infection to a fulminant septic course. Typical symptoms are shivering, fever, myalgia, conjunctival suffusion, vomiting and diarrhea. Icteric and unicteric courses are known. Jaundice is considered as a sign of liver dysfunction but can occur without necrosis of the liver. Antibiotic treatment must be initiated early. Most cases of leptospirosis are described from tropical or subtropical regions. In Germany, approximately 60 cases occur each year [1]. Here, we report the case of a German patient, who presented with acute pancreatitis associated with *Leptospira interrogans* causing fulminant septic shock.

## Case presentation

Five days prior to hospital admission, the 73-year old male suffered from mucous congestion, a swollen throat, fever and shivering. He subsequently developed severe leg-weakness, back pain and jaundice as well as psychiatric symptoms with delusion and hallucination while his fever worsened with a body temperature of 39.8 °C. Myalgia did not occur initially. After consulting his primary care physician, he was immediately transferred to the emergency department (ED) at another hospital.

Apart from a history of depression and *Helicobacter pylori* – gastritis a few years ago, his medical history was unremarkable with no other preexisting conditions. There was no recent travel nor alcohol consumption. The only regular medication was 40 mg of Citalopram daily. The physical examination revealed jaundice and minor petechiae at the lower legs. The abdomen was slightly distended with pain after palpation in the right upper quadrant. Peristaltic sounds were normal. Examination of the heart and the lungs were unremarkable. Conjunctival suffusion was not found.

Initial blood analysis revealed leukocytosis (13.2/nl), thrombocytopenia (23/nl) as well as elevated values for C-reactive protein (CRP, 174.6 mg/l), procalcitonin (PCT, 10.0 ng/ml, cutoff < 0.5 ng/ml), lactate dehydrogenase (LDH) (312 U/l), creatinine (2.95 mg/dl, clearance 20 ml/min/1.73qm), aspartate aminotransferase (AST, 55 U/l), bilirubin (14.9 mg/dl, direct bilirubin 11,72 mg/dl) and lipase (2417 U/l). Sodium (125 mmol/l) and potassium (3.18 mmol/l) were reduced, hemoglobin (Hb), alkaline phosphatase (AP), gamma-glutamyltransferase (GGT) and alanine aminotransferase (ALT) were normal. An overview of selected initial laboratory values can be found in Table 1. Urine analysis revealed nitrite positivity with leukocytes, proteinuria, erythrocytes and bilirubin. An initial X-ray of the chest showed no relevant pathologies.

**Table 1:** Selected initial laboratory values

Leukocytes [4.0–10.0] [/nl]	13.2
Hemoglobin [13.5–17.5] [g/dl]	14.0
Hematocrit [40–53] [%]	42.0
Thrombocytes [140–360] [/nl]	23
Sodium [135–144] [mmol/l]	125
Potassium [3.50–5.10] [mmol/l	3.18
Urea [17–43] [mg/dl]	139
Creatinine [0.7–1.4] [mg/dl]	2.95
AP [40–129] [IU/l]	69
ALT (GPT) [< 45] [IU/l]	36
AST (GOT) [< 35] [IU/l]	55
γ-GT [< 60] [IU/l]	25
Bilirubin [< 1.20] [mg/dl]	14.90
Direct bilirubin [< 0.3] [mg/dl]	11.72
Indirect bilirubin [< 1.20] [mg/dl]	3.18
Lipase [73–393] [IU/l]	2417
LDH [< 248] [IU/l]	312
CRP [< 5] [mg/l]	174.6
PCT [< 0.5] [ng/ml]	10.0

Due to acute septic pancreatitis and acute kidney injury he was transferred to the intensive care unit (ICU). Antibiotic treatment was started with ampicillin/sulbactam. Although no cholestasis or gallstones were found in an initial abdominal ultrasound, a biliary pancreatitis with sepsis was suspected. Thus, an endoscopic retrograde cholangiopancreatography (ERCP) was performed 1 day after his primary presentation at the ED. This procedure revealed a normal duodenum, a swollen papilla vateri and only minor spontaneous biliary drainage was observed. Intrahepatic bile ducts and the common hepatic duct were not dilated. While preparing for the endoscopic procedure, the respiratory and hemodynamic condition of the patient worsened. Consequently he was intubated, commenced on a noradrenaline infusion, and the antibiotic treatment was escalated to meropenem.

A chest CT scan demonstrated bilateral pulmonary infiltrates and small bilateral pleural effusions. Microbiological analysis of blood cultures was negative at all time points tested. A screening for ANA and ANCA was also negative.

The following day, the patient was transferred to our university medical center ICU with sepsis and acute pancreatitis, acute kidney failure and acute respiratory distress syndrome (ARDS).

In our ICU, antibiotic treatment with meropenem and catecholamine treatment with noradrenaline were continued. Blood cultures were taken repetitively and a sample of the tracheal secretion was obtained. After 5 days, weaning from artificial respiration and noradrenaline was possible and the patient was transferred to our gastroenterological ward. The physical examination revealed a slight transient rash of his back. We performed further investigations regarding the etiology of the patient’s condition. A stool test for *Clostridium difficile* antigen was negative. Further sampling by blood cultures, tracheal secretion and urine-analyses showed no growth of any bacteria. A screening for MRSA was negative, as were serologic tests for Hepatits B, Hepatitis C, mumps and Epstein-Barr virus. Acute liver failure with jaundice, hemorrhagic diathesis with purpura and petechiae combined with acute kidney injury accompanied by an odd transient macular, erythematous rash directed to serologic testing for leptospirosis. It revealed strong positive IgG and IgM antibodies. While leptospirosis PCR in the blood was negative, analysis of the tracheal secretion taken during his stay on the ICU revealed the presence of *Leptospira interrogans* DNA. Urine analysis performed at the same time however remained negative for leptospirosis DNA by PCR. PCR was performed by the Institute of Microbiology and Hygiene of the University of Freiburg, Germany, which is accredited by the DAkkS (Deutsche Akkreditierungsstelle, Berlin, Germany) for leptospira PCR for probes of both sterile and non-sterile origin.

**Fig. 1:**
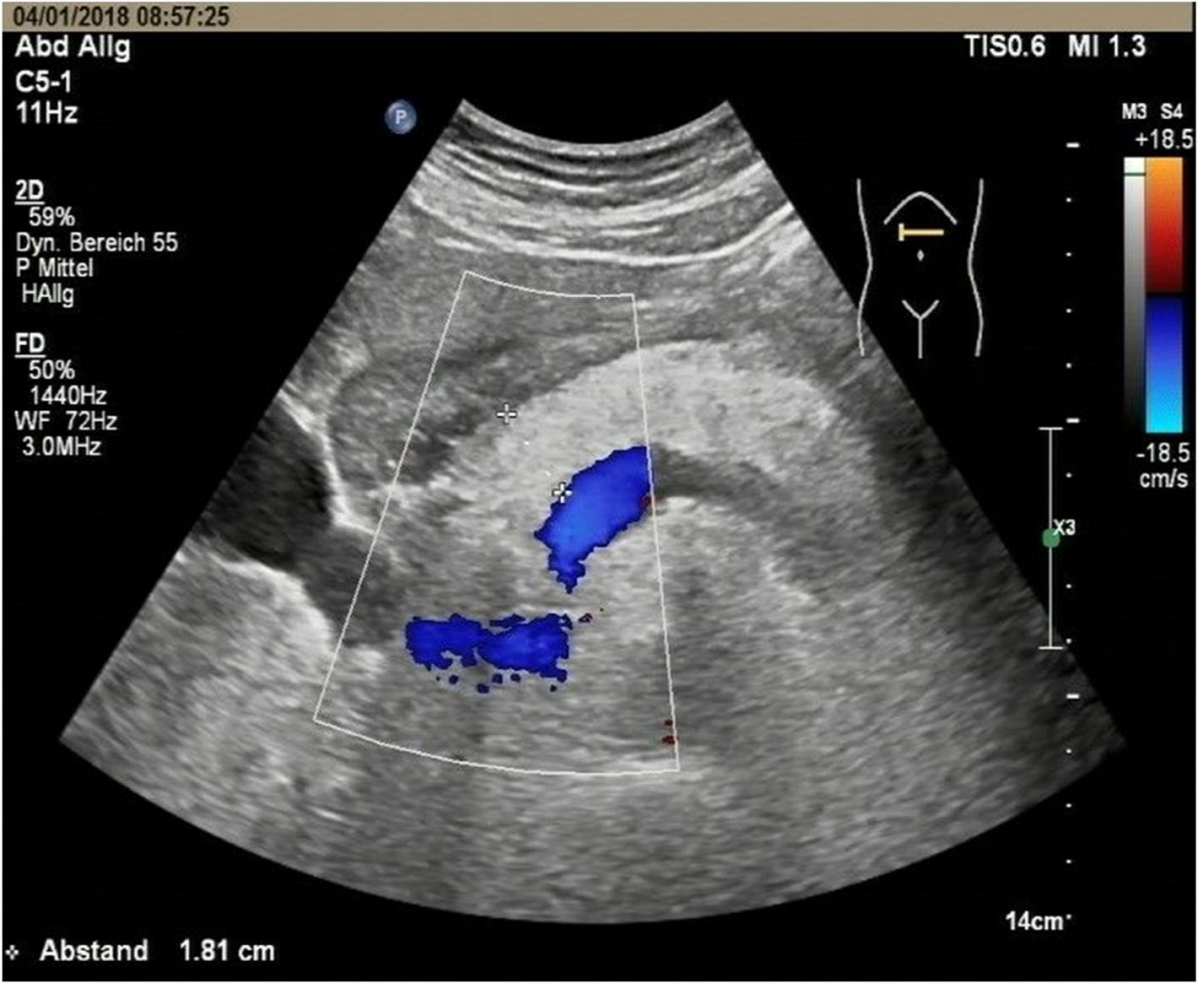
Ultrasound of the patient demonstrating prominent edematous pancreas

An ultrasound of the patient demonstrating prominent edematous pancreas can be found in Fig. 1.

The patient was discharged 18 days after his atypical presentation at the ED with normalized lab values.

## Discussion and conclusion

Here, we report of a patient who presented with acute pancreatitis followed by septic shock with ARDS and acute kidney injury that was caused by leptospirosis. The patient also had mild hepatitis at presentation with clinical jaundice, elevated AST and bilirubin; however, ALT values were not increased throughout the hospital stay and AST levels decreased to normal within 5 days.

The source of his infection eventually remains unclear. There are two relevant possibilities which appear to be most likely: First, the patient lives with several pets including two cats and a dog, which might have shed leptospires in the urine [2]; second, he visited a waste deposal site 7 days before his initial presentation at the ED but had never noticed contact with rodents or recognized a bite injury.

To our knowledge, this is the first case in Central Europe with acute pancreatitis causing vasoplegic shock by leptospirosis. Few cases with leptospirosis have been described in South Europe: One with pancreatitis and cholecystitis in Greece in 2004 [3]. Another case from Greece about a patient from Congo reported pancreatitis in combination with myocarditis, polyarthritis, mononeuritis multiplex and vasculitis with symmetrical peripheral gangrene of the lower extremities [4]. A similar case from Romania was reported in 2013 with necrotizing pancreatitis and severe anemia which required surgical therapy [5]. Some cases with leptospirosis leading to pancreatitis have been described in tropical or subtropical regions [6–18]. An overview of the previously described cases of leptospirosis and pancreatitis can be found in Table 2. However, diagnosis of pancreatitis was often based on amylase-levels [4, 6, 12–14, 18]. These cases have to be interpreted with caution as amylase is often increased in patients with renal failure which is one of the most common manifestations of leptospirosis [19, 20]. Analysis of pancreatic lipase should be preferred as it is more specific for the diagnosis of pancreatitis.

**Table 2:** Overview of previously described cases of leptospirosis and pancreatitis

Case, Country	Diagnosis of leptospirosis	Diagnosis of pancreatitis	ICU/ Operation
Dalamaga M, *J Med*. 2004, Greece [3]	IgM^a^ (ELISA^b^), Microscopic Agglutination Test (MAT^c^), dark-field microscopy of urine	Lipase/Amylase	not reported/not reported
Panagopoulos P, *J Med Case Rep*. 2014, Greece/Congo [4]	IgM^a^, rapid agglutination test for leptospira	Amylase	yes/no abdominal operation
Popa D, J Med Life. 2013, Romania [5]	“test for leptospirosis”	not reported	yes/yes
Herath NJ, *BMC Infect Dis*. 2016, Sri Lanka [6]	Microscopic agglutination test (MAT^c^)	Amylase	yes/not reported
Alian S, *Med J Islam Repub Iran*. 2015, Iran [7]	IFA^d^ (immunofluorescence assay); if the IFA^d^ was negative, MAT^c^(microscopic agglutination test)	Lipase/Amylase	not reported/not reported
Goswami RP, *Trans R Soc Trop Med Hyg*. 2014, India [8]	lateral flow immunoassay by rapid immunochromatographic test (ICT^e^) or ELISA^b^ IgM^a^ or a positive culture	Lipase/Amylase	not reported/not reported
Jain AK, *Indian J Surg*. 2013, India [9]	ELISA^b^	Lipase/Amylase	yes/laparatomy
Baburaj P, *J Assoc Physicians India*. 2008, India [10]	IgM^a^ (ELISA^b^)	Lipase/Amylase	not reported/not reported
Mazhar M, Hawaii J Med Public Health. 2016, USA/Hawaii [11]	IgM^a^	Lipase	yes/not reported
Lim SM, *J Pak Med Assoc*. 2014, Malaysia [12]	IgM^a^	Amylase	not reported/not reported
Yew KL, J Formos Med Assoc. 2015, Malaysia [13]	IgM^a^ by latex agglutination method	Amylase	yes/not reported
Ranawaka N, *BMC Infect Dis*. 2013, Sri Lanka [14]	IgM^a^, Microscopic Agglutination Test (MAT^c^)	Amylase	yes/not reported
Spichler A, Am J Trop Med Hyg. 2007, Brazil [15]	IgM^a^ + IgG^f^ (ELISA^b^)	Lipase/Amylase	yes/no
Silva AP, *Braz J Infect Dis*. 2011, Brazil [16]	IgM^a^ (ELISA^b^)	Lipase/Amylase	not reported/not reported
Kaya E, *World J Gastroenterol*. 2005, Turkey [17]	microagglutination test, dark-field microscopy	Lipase/Amylase	yes/laparatomy
Pai ND, *J Assoc Physicians India*. 2002, India [18]	not reported	Amylase	not reported/not reported
*Our case*	*IgM*^*a*^ *+ IgG*^*f*^*(ELISA*^*b*^*), qPCR*^*g*^	Lipase	*yes/no*

^a^*IgM*: Immunoglobulin M, ^b^*ELISA* Enzyme-linked immunosorbent assay, ^c^*MAT* Microscopic Agglutination Test, ^d^*IFA* immunofluorescence assay, ^e^*ICT* immunochromatographic test, ^f^*IgG* Immunoglobulin G, ^g^*qPCR* real-time quantitative polymerase chain reaction

In Germany, doxycycline, penicillin G, cefotaxime, and ceftriaxone are recommended by the Robert-Koch-Institute as first-line agents for the treatment of leptospirosis. Our patient was initially treated with ampicillin/sulbactam which was then escalated to meropenem. A specific antibiotic treatment like doxycycline, penicillin or ceftriaxone was not administered because of the late diagnosis of leptospirosis.

It is especially peculiar and remarkable that in this patient, acute pancreatitis was the first manifestation of the infection with leptospira followed by septic shock. Commonly, pancreatitis does not represent the initial symptom but is rather observed as a concomitant condition, e.g. in patients with ARDS caused by leptospirosis. Leptospirosis is thought to cause systemic vasculitis in which inflammation of the vascular walls may be a consequence of the direct invasion of the infectious agent or of immune mechanisms such as immune complex deposition, auto-antibodies or cell-mediated immunity [19, 21, 22]. The mechanism of acute pancreatitis in leptospirosis still remains unclear; however, activation of proteolytic enzymes and auto-digestion causing ischemic injury and vasculitis is a probable scenario [10].

Although the reported incidence of pancreatitis in leptospirosis is infrequent, pancreatic involvement may be more common in clinical reality. Thus, due to the fulminant clinical course of our patient with multi-organ failure, we suggest that screening for leptospirosis should be included rather early in the differential diagnosis of acute pancreatitis if other typical causes have been ruled out.

An acute pancreatitis caused by an infection with *Leptospira interrogans* is a very rare but potentially life-threatening disease. Serologic tests and antibiotics for leptospirosis should be considered, if no other reasons for pancreatitis can be identified.

## Abbreviations

ANA: Antinuclear antibody
ANCA: Anti-neutrophil cytoplasmatic antibody
ARDS: Acute respiratory distress syndrome
DNA: Deoxyribonucleic acid
ED: Emergency department
ERCP: Endoscopic retrograde cholangiopancreatography
ICU: Intensive care unit
MRSA: Methicillin-resistant *Staphylococcus aureus*
PCR: Polymerase chain reaction
